# Temperature-Dependent Charpy Impact Toughness and Deformation Mechanisms of Austenitic Fe-32Mn-0.6C Steel

**DOI:** 10.3390/ma18122845

**Published:** 2025-06-17

**Authors:** Jianchao Xiong, Yue Cui, Xin Wang, Caiyi Liu, Silvia Barella, Marco Belfi, Andrea Gruttadauria, Yuhui Wang, Yan Peng, Carlo Mapelli

**Affiliations:** 1 Key Laboratory of Intelligent Equipment Digital Design and Process Simulation, Tangshan University, Tangshan 063000, China; jcxiong@tsc.edu.cn (J.X.); cuiyue@tsc.edu.cn (Y.C.); xinwangysu@163.com (X.W.); 2 National Engineering Research Center for Equipment and Technology of Cold Strip Rolling, Yanshan University, Qinhuangdao 066004, China; liucaiyi@ysu.edu.cn (C.L.);; 3 Department of Mechanical Engineering, Politecnico di Milano, 20156 Milano, Italy; silvia.barella@polimi.it (S.B.); marco.belfi@polimi.it (M.B.); andrea.gruttadauria@polimi.it (A.G.);

**Keywords:** high-manganese steel, impact toughness, cryogenic mechanical properties, plastic deformation

## Abstract

The Charpy impact toughness of single-phase austenitic Fe-32Mn-0.6C steel was systematically investigated across a wide temperature spectrum from 25 °C to −196 °C using Charpy V-notch impact tests. The material exhibited a remarkable temperature dependence of impact energy, decreasing dramatically from 120 J at ambient temperature (25 °C) to 13 J under cryogenic conditions (−196 °C). Notably, a steep transition in impact energy occurred within the critical temperature window of −100 °C to −150 °C. Microstructural analysis revealed that synergistic effects of high strain rates and low temperatures significantly restrict dislocation slip and multiplication mechanisms, while also suppressing deformation twinning activation. This restricted plasticity accommodation mechanism fundamentally differs from the deformation characteristics reported in conventional low-carbon high-manganese steels and other face-centered cubic (FCC) alloy systems.

## 1. Introduction

In response to the global effort to reduce carbon dioxide emissions, the development of clean energy sources such as liquid hydrogen and liquefied natural gas has gained significant momentum. This shift has driven an increasing demand for high-performance and cost-effective cryogenic alloys. High-manganese austenitic steels have emerged as promising candidates due to their high tensile strength, excellent work-hardening capability, and relatively low cost [1,2,3,4]. Furthermore, through compositional design and microstructural control, the strength and ductility of high-manganese austenitic steel can be simultaneously enhanced as the temperature decreases. This is attributed to the primary plastic deformation mechanism transitioning from dislocation slip at room temperature (RT) to deformation twinning or phase transformations and dislocation slip at liquid nitrogen temperature (LNT) [5,6]. Therefore, high-manganese austenitic steel has become a focal point in the current research on cryogenic alloys.

Previous studies have primarily focused on the tensile properties and deformation mechanisms of cryogenic high-manganese steels with varying compositions and microstructures [4,7]. However, research on the impact properties of high-manganese steels at low temperatures is limited [8,9]. Impact performance is crucial for the application of high-manganese steels in cryogenic environments [10]. Existing studies have reported on the influence of microstructure on the impact performance of high-manganese steels. It has been observed that reducing the grain size of 0.2% C-Mn steel (low-carbon ferritic steel) can lower the ductile-to-brittle transition temperature (DBTT), attributed to the combined effects of smaller ferrite grain size and delamination. The finer ferrite grain size contributes to increased strength and toughness, while delamination reduces the triaxiality of the stress state during impact testing, hindering brittle fracture and thus lowering the DBTT [11]. In high-manganese steels, solution treatment has been reported to enhance Charpy impact toughness at –196 °C, with the fracture mode remaining ductile and mechanical twinning identified as the primary deformation mechanism [12]. Kang et al. [13] reported that Fe-35Mn-6Al-0.1C (wt.%) steel exhibits high-impact energies of 331 J at room temperature and 254 J at cryogenic temperature. Limited twinning activity at low temperature contributed little to toughness, while crack initiation at the γ/α interface was identified as the primary cause of impact energy reduction. Shi et al. [14] reported that the impact energy of a Fe_62_Co_5_Ni_10_Cr_13_Si_7_Al_3_ medium-entropy alloy decreases from 95.5 J at 298 K to 32.2 J at 77 K. This deterioration was attributed to the reduced plastic zone ahead of the crack tip and a transition in fracture mode from ductile dimple rupture to a mixed dimple–cleavage mechanism, both of which limit energy dissipation during crack propagation.

Austenitic steels typically do not exhibit a pronounced ductile-to-brittle transition phenomenon [15], as seen in Fe-30Mn-0.05C steel [16], Fe-30Mn-0.11C steel [6], and various austenitic stainless steels [17,18]. Notably, Fe-30Mn-0.11C steel with an average grain size of 5.6 μm demonstrates a Charpy impact energy of 453 ± 4 J at −196 °C [6]. However, study on the low-temperature impact properties and deformation mechanisms of high-carbon high-manganese steels is limited. It has been reported that during quasi-static tensile testing at −196 °C, Fe-32Mn-0.6C steel can activate deformation twinning at relatively low strains due to high stress, low stacking fault energy (SFE), and high carbon content, leading to a high work-hardening rate and premature fracture [19]. This study systematically investigates the impact performance and deformation mechanisms of Fe-32Mn-0.6C steel over the temperature range of 25 °C to −196 °C, revealing a pronounced ductile-to-brittle transition in Fe-32Mn-0.6C steel.

## 2. Materials and Methods

The steel selected for the experimental study is a single-phase high-manganese austenitic steel (Fe-32Mn-0.6C) with an average grain size of 16.9 ± 4.1 μm, as determined by Electron Backscatter Diffraction (EBSD) analysis. The detailed chemical composition, microstructure, and preparation process are available in reference [19]. The EBSD characterization was carried out with a Zeiss Sigma 500 scanning electron microscope (SEM) (Oberkochen, Germany) equipped with an Oxford C-Nano detector (Oxfordshire, UK). Fractography analysis was conducted using a FEI-Scios field emission scanning electron microscope (SEM) (Hillsboro, OR, USA). The SEM operating voltage was set at 10 kV, while the operating voltage for EBSD was 20 kV. EBSD samples were prepared by wire electrical discharge machining, followed by a standard mechanical polishing procedure to remove surface machining marks and scratches. Subsequently, electro-polishing was performed in a solution of 10% HClO_4_ and 90% C_2_H_5_OH (volume fraction) at a polishing voltage of 30 V for 30 s at 25 °C. The EBSD scan step size was 0.6 μm.

Room- and low-temperature Charpy impact tests were conducted using an MTS impact testing machine equipped with a cryogenic chamber. Impact specimens were cut from the experimental steel plate by wire electrical discharge machining, followed by milling to remove surface machining marks. Half-size Charpy V-notch specimens with dimensions of 5 mm (thickness) × 10 mm (width) × 55 mm (length) were used in this study. The length direction of the specimens was parallel to the rolling direction of the steel plate. The V-notch was machined on the transverse direction (TD) plane of the specimen. The V-notch radius was 0.25 ± 0.025 mm. For low-temperature impact testing, specimens were soaked for more than 30 min in a cryogenic apparatus set to the designated temperatures (0 °C, −25 °C, −50 °C, −75 °C, −100 °C, and −150 °C) or in liquid nitrogen (−196 °C) to ensure that the specimens reached the specified temperatures. The specimens were then quickly removed using tongs and immediately subjected to impact testing [20]. For each temperature condition, a total of three Charpy V-notch specimens were tested to ensure data reliability, and the average impact energy was reported. The lateral expansion values of the specimens after impact were measured according to GB/T 229-2020 [20].

## 3. Results

### 3.1. Effect of Temperature on Charpy Impact Properties

**Figure 1:**
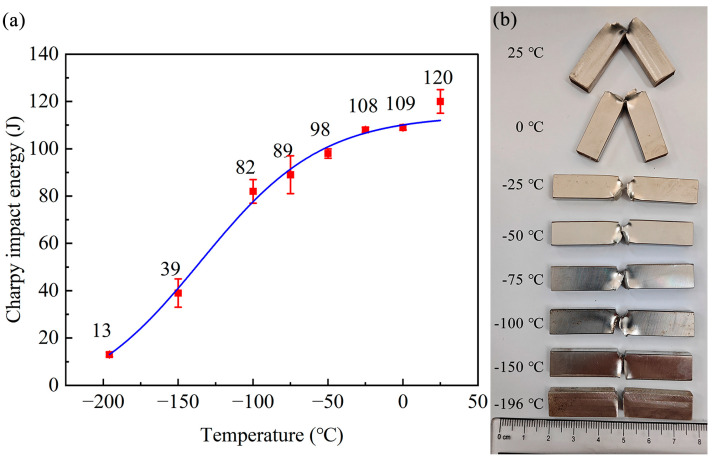
Impact behavior of Fe-32Mn-0.6C steel at different temperatures: (**a**) variation in impact energy with temperature; (**b**) fracture appearance of specimens after impact testing at various temperatures.

Figure 1a shows the Charpy impact energy of Fe-32Mn-0.6C steel with an average grain size of 16.9 μm at various testing temperatures. It is evident that the impact energy of Fe-32Mn-0.6C steel is significantly temperature-dependent. As the testing temperature decreases from 25 °C to −196 °C, the impact energy of Fe-32Mn-0.6C steel decreases from 120 J to 13 J. In the temperature range from 25 °C to −100 °C, the impact energy decreases gradually, dropping from 120 J at 25 °C to 82 J at −100 °C, a reduction of 32%. Below −100 °C, the impact energy decreases rapidly. At −196 °C, the impact energy is only 13 J, representing an 89% decrease compared to the impact energy measured at 25 °C. Table 1 shows the lateral expansion values of the specimens after impact testing at different temperatures. According to GB/T 229-2020, when the absorbed energy reaches 50% of the upper shelf energy or the lateral expansion value decreases to 0.9 mm, the corresponding temperature marks a significant change in the material’s impact performance. Based on these criteria, the impact properties of Fe-32Mn-0.6C steel exhibit a notable decline in the temperature range of −100 °C to −150 °C.

**Figure 2:**
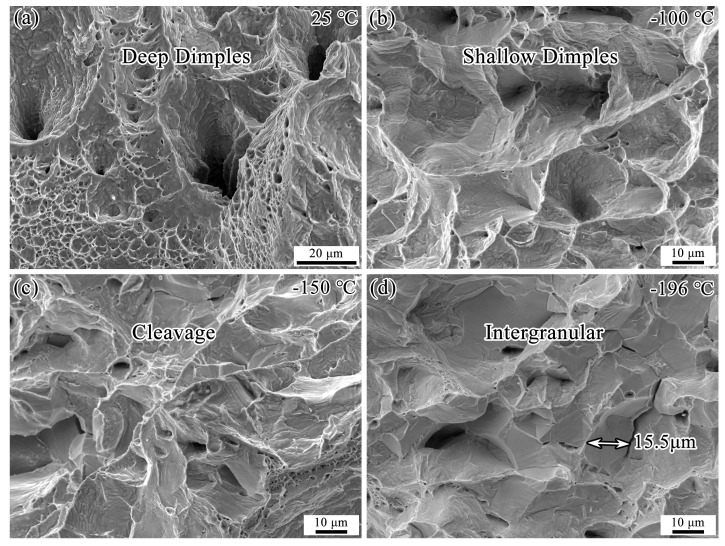
Fracture surface micrographs of Fe-32Mn-0.6C steel after impact testing at (**a**) 25 °C, (**b**) −100 °C, (**c**) −150 °C, and (**d**) −196 °C.

Figure 1b shows the photographs of Fe-32Mn-0.6C steel specimens after impact testing at different temperatures. As the testing temperature decreases, the degree of plastic deformation of the specimens gradually reduces, corresponding to the trend observed in impact energy. The specimen impacted at 25 °C did not fracture completely, indicating crack blunting at this temperature. Conversely, the fracture surface of the specimen impacted at −196 °C is almost flat, indicating rapid crack propagation leading to fracture. Furthermore, the fracture surface morphologies of the specimens impacted at 25 °C, −100 °C, −150 °C, and −196 °C were examined, as shown in Figure 2. The fracture surface of the specimen impacted at 25 °C exhibits large and deep dimples, indicative of a typical ductile fracture mode. At −100 °C, the fracture surface shows shallow dimples. At −150 °C, the specimen exhibits cleavage fracture. At −196 °C, the fracture mode transitions to intergranular fracture. The size of the intergranular fracture facets is close to the average grain size of the specimen: 16.9 μm. These results indicate that as the testing temperature decreases, the fracture mode of Fe-32Mn-0.6C steel transitions from ductile to brittle.

### 3.2. Effect of Temperature on Charpy Impact Deformation Mechanisms

**Figure 3:**
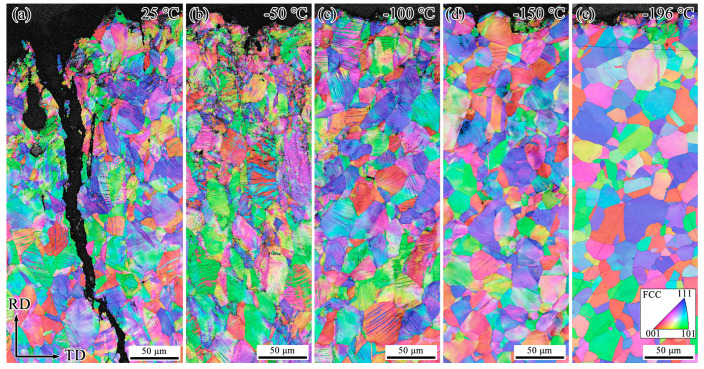
EBSD IPF maps near the fracture surface of Fe-32Mn-0.6C steel after impact testing at different temperatures. (**a**) 25 °C, (**b**) −50 °C, (**c**) −100 °C, (**d**) −150 °C, (**e**) −196 °C.

**Figure 4:**
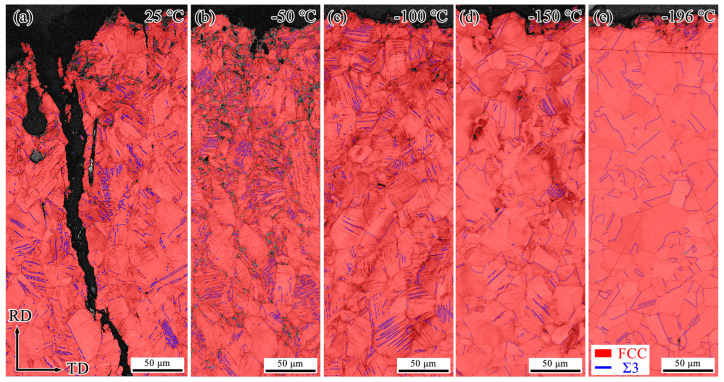
EBSD Phase maps near the fracture surface of Fe-32Mn-0.6C steel after impact testing at different temperatures. (**a**) 25 °C, (**b**) −50 °C, (**c**) −100 °C, (**d**) −150 °C, (**e**) −196 °C.

**Figure 5:**
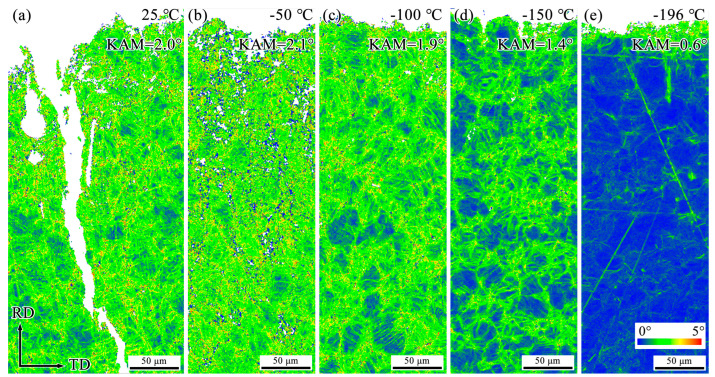
EBSD KAM maps near the fracture surface of Fe-32Mn-0.6C steel after impact testing at different temperatures. (**a**) 25 °C, (**b**) −50 °C, (**c**) −100 °C, (**d**) −150 °C, (**e**) −196 °C.

To investigate the primary reasons for the decrease in impact energy of Fe-32Mn-0.6C steel at low temperatures, EBSD analysis was conducted on the microstructures of specimens after impact testing at different temperatures. Figure 3, Figure 4 and Figure 5 show the Inverse Pole Figure (IPF), Phase, and Kernel Average Misorientation (KAM) maps of the regions near the fracture surfaces of Fe-32Mn-0.6C steel specimens impacted at various temperatures. For comparative analysis, the characterization was performed at the middle position near the fracture surface of each specimen.

It is well-established that Σ3 Coincidence Site Lattice (CSL) boundaries are characteristic of annealing twins and deformation twins in FCC metals. At 25 °C, secondary cracks perpendicular to the main crack and numerous deformation twins were observed near the fracture surface, as shown in Figure 3 and Figure 4. The formation of secondary cracks and deformation twins consumes a significant amount of energy and impedes crack propagation, contributing to higher impact energy [21]. Although the identification rate of deformation twin boundaries was relatively low due to the EBSD step size being set to 0.6 μm, the comparative analysis still reveals that the fraction of deformation twin boundaries decreases as the testing temperature decreases. After impact fracture at −196 °C, deformation twins were only observed near the fracture surface (see Figure 4). As the distance from the fracture surface increased, the amount of deformed structure significantly decreased, indicating that plastic deformation at −196 °C primarily occurred near the fracture surface. Figure 4 shows that no martensite formation was detected in the deformed structures after impact at different temperatures. Figure 5 demonstrates that as the testing temperature decreases from 25 °C to −196 °C, the KAM values of the specimens gradually decline, indicating a reduction in plastic deformation near the fracture surface.

## 4. Discussion

The results above indicate that as the testing temperature decreased from 25 °C to −196 °C, the Charpy impact energy of Fe-32Mn-0.6C steel significantly decreased, with a notable reduction in impact performance observed between −100 °C and −150 °C. As the testing temperature decreased, the plastic deformation capability of Fe-32Mn-0.6C steel decreased. Typically, austenitic steels do not exhibit a distinct ductile-to-brittle transition temperature, as observed in stainless steels [18] and Fe-30Mn-0.11C steel [6]. It is generally believed that the martensitic phase transformation at low temperatures is a major factor contributing to the reduced impact energy in high-manganese steels [6,10,22]. However, as shown in Figure 4, no martensite formation was observed after impact fracture at different temperatures. This is attributed to the presence of significant austenite-stabilizing elements such as Mn and C in Fe-32Mn-0.6C steel, promoting a stable austenitic phase. The SFE values of Fe-32Mn-0.6C steel at 25 °C and −196 °C are 45.36 mJ/m^2^ and 40.82 mJ/m^2^, respectively, falling within the Twinning-Induced Plasticity (TWIP) effect range [23]. The detailed calculation procedure can be found in our previous work [6]. Therefore, the significant decrease in impact energy below −100 °C is not associated with martensitic phase transformation. Furthermore, previous study has not detected the formation of carbides in Fe-32Mn-0.6C steel, thus excluding any negative effects of carbides on impact properties [19].

It has been reported that Fe-32Mn-0.6C steel exhibits premature failure at −196 °C during quasi-static tensile testing due to rapid activation of deformation twins at lower strains, ultimately forming a multi-stage twinning grid structure [19]. In contrast, in the current high strain rate impact tests, the interaction of high strain rate and low temperature leads to a significant reduction in the quantity of deformation twins near the fracture surface (see Figure 3). The low temperature tends to decrease the stacking fault energy slightly and affect dislocation mobility, while the high strain rate reduces the time available for twin nucleation and growth, thus suppressing deformation twinning during impact. As shown in Figure 5, the average *KAM* value of Fe-32Mn-0.6C steel decreases gradually as the testing temperature decreases. The *KAM* value typically correlates with the density of geometrically necessary dislocations (*GNDs*) in deformed alloys, which can be approximately calculated using the following equation [24]:

(1) $$\rho_{GND}=\frac{{2KAM}_{ave}}{\mu b}$$

where $\rho_{GND}$ is the *GND* density, *μ* (0.6 μm) is the unit length, and *b* = 0.253 nm is the magnitude of Burgers vector.

**Figure 6:**
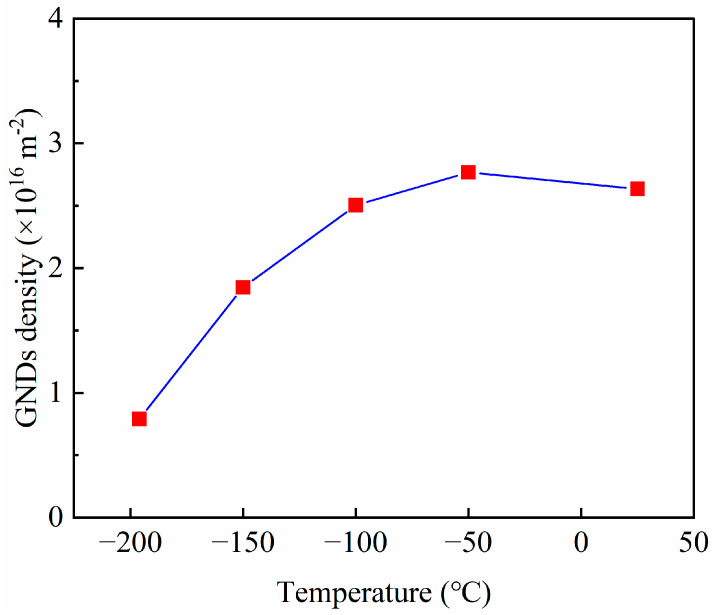
Geometrically necessary dislocation density near the fracture surface of Fe-32Mn-0.6C steel after impact testing at different temperatures. The red squares are data points, and the blue solid line connects these data points.

In this study, the *GND* density near the fracture surface at different temperatures was quantitatively calculated using Equation (1) based on the average *KAM* values. Figure 6 shows *GND* density near the fracture surface of Fe-32Mn-0.6C steel after impact testing at different temperatures. The results indicate that as the temperature decreases, the GND density near the fracture surface significantly decreases, primarily due to the increased lattice friction stress at lower temperatures, which hinders dislocation slip and multiplication [25]. On the other hand, the high strain rate during impact testing may also affect dislocation activities by reducing the time available for dislocation motion and twin nucleation. The formation of deformation twins requires high dislocation density and localized high stress. Due to the reduced density of partial dislocations, mainly resulting from the combined effects of low temperature and high strain rate, nucleation sites for deformation twins decrease, thereby inhibiting twin formation [23]. Deformation twins are critical microstructures for enhancing the plasticity and toughness of high-manganese steels, as they can store dislocations and blunt cracks [21,26]. The difficulty in dislocation slip and multiplication, along with the reduction in deformation twinning, are the primary reasons for the decreased impact toughness of Fe-32Mn-0.6C steel at low temperatures. This phenomenon contrasts with the findings of Shen et al. [27], where high strain rates and lower temperatures promoted the formation of ε-martensite in Fe-25Mn-3Al-3Si steel, enhancing low-temperature toughness. The observed differences may be attributed to variations in steel composition and SFE.

It has been reported that fully recrystallized Fe-30Mn-0.11C steel exhibits an impact toughness of 269–453 J at −196 °C (tested using full-size Charpy specimens) [6]. Similarly, fully recrystallized Fe-30Mn-0.05C steel shows an impact toughness of 172–325 J at −196 °C (tested using full-size Charpy specimens) [16]. Both steels exhibit extensive deformation twins after impact at −196 °C, contributing to their work hardening and high impact toughness. In contrast, Fe-32Mn-0.6C steel demonstrates an impact toughness of only 13 J at −196 °C (tested using half-size Charpy specimens). It is worth noting that half-size specimens generally exhibit lower absolute impact energy than full-size specimens due to their smaller volume and reduced fracture surface area. Therefore, while the absolute values are not directly comparable, the comparison here primarily aims to highlight the differences in deformation mechanisms and trends associated with alloy composition. The significant differences in low-temperature impact toughness among Fe-30Mn-0.11C, Fe-30Mn-0.05C, and Fe-32Mn-0.6C steels may be attributed to their carbon content. It has been shown that the yield strength of high-carbon steels is more sensitive to changes in testing temperature compared to low-carbon steels [19,28]. As temperature decreases, the yield strength of high-carbon steels significantly increases due to enhanced lattice friction at low temperatures. This substantial increase in low-temperature strength can lead to a reduction in their low-temperature impact toughness.

It should be noted that the spatial resolution of EBSD used in this study limits the ability to capture extremely fine deformation features such as narrow deformation twins or highly localized dislocation structures. Additionally, direct in situ observation of the cryogenic impact process remains extremely challenging due to the combination of high strain rate and low temperature, which restricts real-time microstructural analysis [29]. These methodological limitations may result in an underestimation of the extent of local plastic deformation. Therefore, future studies could incorporate complementary modeling approaches, such as crystal plasticity finite element simulations or molecular dynamics methods, to provide deeper insights into the deformation mechanisms and to validate the present experimental findings [30,31].

## 5. Conclusions

The Charpy impact tests on single-phase austenitic Fe-32Mn-0.6C steel demonstrates a distinctive cryogenic degradation mechanism governed by temperature-dependent deformation constraints. A three-stage toughness evolution is observed: (i) gradual energy reduction from 120 J at 25 °C, (ii) accelerated decline through the critical transition regime (−100 °C to −150 °C), and (iii) ultimate low-energy fracture at −196 °C (13 J). This sequential embrittlement directly correlates with the progressive restriction of strain accommodation mechanisms, where cryogenic conditions synergistically impede dislocation glide dynamics and suppress deformation twinning activation under impact loading. Such dual suppression of plasticity pathways establishes a unique failure paradigm distinct from conventional FCC alloys, providing critical insights for cryogenic steel design.

## Figures and Tables

**Table 1:** Lateral expansion values of specimens after Charpy impact testing at different temperatures.

**Testing temperature (°C)**	25	0	−25	−50	−75	−100	−150	−196
**Lateral expansion (mm)**	2.30 ± 0.16	1.70 ± 0.13	1.68 ± 0.11	1.72 ± 0.10	1.45 ± 0.10	1.43 ± 0.09	0.89 ± 0.08	0.49 ± 0.09

## Data Availability

The original contributions presented in the study are included in the article, further inquiries can be directed to the corresponding author.
